# Determination of Digestible Indispensable Amino Acid Scores for Plant Proteins and Skim Milk Powder Measured in Pigs

**DOI:** 10.3390/ani15050650

**Published:** 2025-02-24

**Authors:** Junghyun Oh, Noa Park, Beob Gyun Kim

**Affiliations:** Department of Animal Science, Konkuk University, Seoul 05029, Republic of Korea; wjdgus113@konkuk.ac.kr (J.O.); noa0227@konkuk.ac.kr (N.P.)

**Keywords:** food ingredients, ileal digestibility, protein quality, swine

## Abstract

The objective was to determine the digestible indispensable amino acid score (DIAAS) of food sources using the pig model and in vitro procedures. The standardized ileal digestibility (SID) of amino acids (AA) in white rice, wheat, pea protein concentrate (PPC), soy protein isolate (SPI), and skim milk powder (SMP) were determined in pigs. Twelve barrows with an initial body weight of 28.4 kg (standard deviation = 4.0) equipped with a T-cannula at the distal ileum were allotted to a replicated 6 × 5 incomplete Latin square design with six diets and five periods. Five experimental diets contained each ingredient as the only source of AA. Additionally, a nitrogen-free diet was prepared to measure the basal endogenous losses of AA for the calculation of the SID of AA. The in vitro ileal disappearance (IVID) of crude protein (CP) in the five food sources was also determined using the conventional flask procedure and the multi-sample simultaneous in vitro procedure employing the Daisy^II^ incubator. The DIAAS values for white rice were 42, 50, and 60 for infants, children, and adults, respectively. For wheat, the DIAAS values were 46, 56, and 66, respectively. The DIAAS values for PPC were 47, 58, and 69 for infants, children, and adults, respectively. For SPI, the values were 61, 74, and 87, respectively. The DIAAS for SMP were 82, 112, and 131 for infants, children, and adults respectively. The IVID of CP in SMP was greater (*p* < 0.05) than that in other sources. The DIAAS calculated based on IVID of CP were similar to those determined based on the SID of AA in pigs. In conclusion, white rice, wheat, pea protein concentrate, and soy protein isolate were observed to have DIAAS values less than 100, indicating that these ingredients need to be combined with other protein sources to meet the indispensable AA requirements. Skim milk powder had a DIAAS greater than 100 for children and adults.

## Simple Summary

The digestible indispensable amino acid scores employing the pig model are widely used to determine the protein quality of human food sources. In this study, the standardized ileal digestibility of amino acids was measured for white rice, wheat, pea protein concentrate, soy protein isolate, and skim milk powder in pigs. Additionally, the in vitro ileal disappearance of protein was determined using the flask and the Daisy^II^ procedures. Similar digestible indispensable amino acid scores for food sources were observed among the methods using the standardized ileal digestibility of amino acids and in vitro procedures. The digestible indispensable amino acid scores for white rice, wheat, pea protein concentrate, and soy protein isolate were less than 100 for infants, children, and adults, whereas those for skim milk powder were greater than 100 for children and adults based on the ileal amino acid digestibility in pigs. The in vitro ileal disappearance procedures also resulted in similar digestible indispensable amino acid scores. Overall, the protein quality of skim milk powder has greater protein quality compared with white rice, wheat, pea protein concentrate, and soy protein isolate. Additionally, in vitro ileal crude protein disappearance can be used as a reference for the digestible indispensable amino acid score of food sources.

## 1. Introduction

According to the FAO, the demand for food is increasing along with population growth [1]. To meet the nutritional needs of the increasing global population in the world, it is important to define the nutritional quality of foods consumed by humans accurately. Among nutrients in foods, protein is one of the most fundamental components, which constitutes tissues in the human body [2]. Protein undernutrition results in stunted growth, disease, and early death [1,3]. An adequate supply of protein is necessary to fulfill the amino acid (AA) requirements of humans, and thus, an accurate evaluation of foods should precede identifying the quantity of digestible AA [3].

The digestible indispensable amino acid score (DIAAS) is a recent approach for evaluating the protein quality of foods intended for human consumption [1,4]. The DIAAS is considered superior to the previous method, the protein digestibility-corrected amino acid score (PDCAAS), which is based on the assumption that crude protein (CP) and all AA have the same digestibility. In addition, although AA absorption primarily occurs in the small intestine and AA passing into the hindgut undergo transamination or deamination due to fermentation by microorganisms, the PDCAAS adopts the total tract digestibility of CP using rats [5,6], which causes an inaccurate evaluation of AA digestibility [7]. However, the DIAAS is obtained using the standardized ileal digestibility (SID) of individual AA using the pig model, which is considered an appropriate model for human digestive physiology due to its similarity to humans in terms of digestive systems and the comparable SID of AA [8,9]. For this reason, the DIAAS is recommended by the FAO for its more precise measurement of protein quality by assessing the digestible indispensable AA based on the growing pig model, which is an excellent alternative approach when conducting experiments employing humans as subjects is not feasible [1].

In vitro assays have been utilized to estimate protein digestibility in feed ingredients for pigs [10] based on the high correlation between in vitro and in vivo nutrient digestibility [11]. In vitro assays are less labor-intensive and more cost-effective compared with animal experiments. A conventional in vitro assay is available [11], and the multi-sample simultaneous in vitro procedure using the Daisy^II^ incubator (ANKOM Technology, Macedon, NY, USA) was developed in a previous study [12] to determine the digestibility of feed ingredients for pigs. Given that protein digestibility is highly correlated with AA digestibility, the SID of AA could potentially be replaced with the fixed value of IVID of CP for all AA in calculating the DIAAS in food sources. However, data comparing DIAAS in food sources based on the SID of AA and the IVID of CP are not available. Therefore, the present study aimed to determine the SID of AA, the IVID of CP using the conventional flask method (IVID_Flask_ of CP), and the IVID of CP using the Daisy^II^ incubator (IVID_Daisy_ of CP) in various food protein sources including white rice, wheat, pea protein concentrate (PPC), soy protein isolate (SPI), and skim milk powder (SMP) in pigs and to compare the DIAAS among the methods derived from in vivo and in vitro methods.

## 2. Materials and Methods

The procedures for the animal experiment were approved by the Institutional Animal Care and Use Committee of Konkuk University (Seoul, Republic of Korea, KU23172).

### 2.1. Animals, Diets, and Experimental Design

Twelve barrows (Landrace × Yorkshire) with an initial body weight (BW) of 28.4 kg (standard deviation = 4.0) were equipped with a T-cannula (5 cm barrel length and 1.3 cm inner diameter) at the distal ileum [13]. The pigs were individually raised in pens (1.2 m × 1.6 m), and a feeder and a nipple drinker were installed in each pen. The five test ingredients consisted of white rice, wheat, PPC, SPI, and SMP (Table 1). Five experimental diets contained 96.8% white rice, 96.9% wheat, 25.0% PPC, 20.0% SPI, and 50.0% SMP as the sole source of AA (Table 2 and Table 3). A nitrogen-free diet was also prepared to determine the basal endogenous losses (BEL) of AA for the calculation of the SID of AA. All experimental diets contained 0.5% chromic oxide as an indigestible index. Vitamins and minerals were added to the experimental diets to meet or exceed the requirement estimates of the NRC [14]. After a 12-day recovery period from surgery, the pigs were allocated to a replicated 6 × 5 incomplete Latin square design with six diets and five periods. Potential carryover effects were minimized using a spreadsheet-based program [15].

### 2.2. Feeding and Ileal Digesta Collection

The daily feed allotments were calculated as 4.0% of the initial BW and provided in two equal meals at 08:00 and 17:00 h. The daily feed allowance was recalculated at the beginning of each period based on BW. Water was available to the animals at all times. The initial 6 days of each period were considered the adaptation period of the experimental diet, followed by a 2-day collection of ileal digesta samples from 08:30 to 17:00 h. A plastic bag with wire (Whirl-Pak bag; NASCO, Fort Atkinson, WI, USA) was attached to the T-cannula barrel for the ileal digesta collection. Bags were replaced whenever the plastic bag was filled with ileal digesta, or at least once every 30 min. The collected ileal digesta samples were immediately stored at −20 °C.

### 2.3. Conventional Two-Step In Vitro Procedure

A conventional two-step in vitro assay was performed to measure the IVID_Flask_ of CP in white rice, wheat, PPC, SPI, and SMP [11]. During the first step of the procedure, 1 g of the ingredient in a 100-mL conical flask was digested using a pepsin solution (10 mg/mL; 250 units/mg solid, P7000, pepsin from porcine gastric mucosa; Sigma-Aldrich, St. Louis, MO, USA) under pH 2.0 for 6 h at 39 °C. During the second step, a pancreatin solution (50 mg/mL; 4 USP, P1750, pancreatin from porcine pancreas; Sigma-Aldrich, St. Louis, MO, USA) was added to the flask for incubation at pH 6.8 for 18 h at 39 °C. Undigested residues were collected for the analysis of CP.

### 2.4. Multi-Sample Simultaneous In Vitro Procedure

Based on the two-step conventional in vitro method using a flask described by Boisen and Fernández [11] and the three-step in vitro method using a Daisy^II^ incubator described by Son [12], a two-step in vitro procedure utilizing the Daisy^II^ incubator was conducted. In the first step of the procedure, 1 g of the ingredient in a R55 filter bag (ANKOM Technology, Macedon, NY, USA) was digested using a pepsin solution (5 mg/mL) under pH 2.0 for 3 h at 39 °C. In each jar, 11 bags consisting of 2 bags for each of the five ingredients and 1 blank bag were placed. In the second step, a pancreatin solution (37.5 mg/mL) was added to the sample in the flask for incubation at pH 6.8 for 9 h at 39 °C. Undigested residues were collected for the analysis of CP. The total volume of solutions in a jar was calculated by multiplying the volume of solutions per sample in the conventional procedure [11] by the number of sample bags [16]. At the end of the procedure, all bags were transferred to a beaker for rinsing twice with distilled water in a shaking incubator (LSI-3016R; Daihan Labtech, Namyangju, Republic of Korea) for 3 min. Then, the bags were rinsed twice with 96% ethanol and 99.5% acetone for 5 min each. Lastly, the bags were pat-dried with paper towels (Wypall L25^®^, Yuhan-Kimberly, Seoul, Republic of Korea) and further dried in a forced-air drying oven to be dried at 80 °C for 24 h. The procedures were triplicated using three jars.

### 2.5. Chemical Analyses

Before the chemical analyses, ileal digesta samples were freeze-dried and finely ground. The gross energy of all food ingredients and diets was determined using a Parr 6400 calorimeter (Parr Instruments Co., Moline, IL, USA). Dry matter (method 930.15), CP (method 990.03), ether extract (method 920.39), and ash (method 942.05) in the ingredient and diet samples were analyzed following the AOAC [17]. Additionally, undigested residues from in vitro assays were analyzed for CP by the AOAC methods [17]. The AA concentrations in the food ingredients, experimental diets, and ileal digesta were also analyzed by the AOAC methods (method 982.30) [17]. The procedure described by Fenton and Fenton [18] was used to analyze the chromium (Cr) concentrations present in diets and ileal digesta samples.

### 2.6. Calculations and Statistical Analyses

The apparent ileal digestibility (AID), BEL, and SID of CP and AA were calculated based on equations reported previously [19,20]. The IVID of CP was calculated using previously reported equations [10]. The concentration of SID AA (g/kg) in each diet was determined by multiplying the SID value (%) of each AA by its concentration (g/kg) in the diet. The concentration of SID AA was then divided by the concentration of CP in the diet to obtain the digestible indispensable AA content (mg) in 1 g of protein. Additionally, the DIAAS determined using in vitro procedures was calculated by replacing the SID of AA with the IVID of CP in the equation. The digestible indispensable AA-to-AA in the reference protein ratio was calculated [1]:

Digestible indispensable AA-to-AA in the reference protein ratio (%) = digestible indispensable AA content (mg) in 1 g CP of food ÷ the same dietary indispensable AA (mg) in 1 g of the reference protein × 100%

The ratios were calculated using the reference protein based on the FAO [1] for birth to 6 months, children from 6 months to 3 years, children older than 3 years, adolescents, and adults. The DIAAS was calculated using the following equation:

DIAAS (%) = lowest value of digestible indispensable AA-to-AA in the reference protein ratio

The data were analyzed using the MIXED procedure of SAS 9.4 (SAS Inst. Inc., Cary, NC, USA). Outliers were defined as values exceeding 1.5 times the interquartile range beyond either the first or third quartiles. In the statistical model, the experimental diet was a fixed variable, and the replication, period within replication, and animal within replication were random variables. The GLM procedure in SAS was used to analyze data for the IVID of CP. The test ingredient was a fixed variable in the model. Least squares means of each treatment were calculated, and differences among them were evaluated using the SAS PDIFF option with Tukey’s adjustment. The experimental unit in the animal experiment was the pig, whereas the flask or the jar was the experimental unit in the in vitro procedures. The statistical significance was declared at an alpha level of 0.05.

## 3. Results

A total of six outliers were identified, with one outlier detected in each group fed wheat, PPC, SPI, and SMP diets and two outliers detected in the group fed the white rice diet. The concentrations of CP among the ingredients ranged from 7.5% to 84.2% on an as-is basis (Table 1).

The AID of CP in PPC, SPI, and SMP was greater (*p* < 0.05) than that in white rice and wheat (Table 4). The AID of all indispensable AA except for Met and Trp in PPC, SPI, and SMP was greater (*p* < 0.05) than that in white rice and wheat.

The SID of CP in white rice, PPC, SPI, and SMP was greater (*p* < 0.05) than that in wheat (Table 5). The SID of most indispensable AA in SMP was greater (*p* < 0.05) than that in other sources except for His, Ile, Lys, Thr, Trp, and Val.

The IVID_Flask_ of CP in white rice and SMP was greater (*p* < 0.05) than that in wheat, PPC, and SPI (Table 6). The IVID_Daisy_ of CP in SPI and SMP was greater (*p* < 0.05) than that in white rice, wheat, and PPC.

Based on the SID of AA, the DIAAS for white rice was 42 (Lys), 50 (Lys), and 60 (Lys) for infants, children, and adults, respectively (Table 7). The DIAAS was 46 (Lys), 56 (Lys), and 66 (Lys) for wheat, 47 (Trp), 58 (SAA), and 69 (SAA) for PPC, 61 (SAA), 74 (SAA), and 87 (SAA) for SPI, and 82 (SAA), 112 (SAA), and 131 (SAA) for SMP in infants, children, and adults, respectively. The DIAAS based on the SID of AA was similar to the DIAAS based on the IVID_Flask_ and IVID_Daisy_ of CP in the test ingredients.

## 4. Discussion

White rice, wheat, PPC, SPI, and SMP are foods commonly consumed by humans or raw materials used for producing food products. Cereal grains and legume proteins contribute to daily energy and protein intake for humans [9], and dairy products are widely consumed due to their relatively high digestible nutrient contents compared with cereal grains [6]. For this reason, an approach of combining dairy products with cereals can supply sufficient AA to fulfill human requirements [21]. To make the approach feasible, determining protein quality and digestible AA in food sources is important in enhancing human food databases and aiding in formulating adequate protein diets for humans. The DIAAS has been suggested as the best method for determining protein quality in food proteins based on the SID of AA in pigs obtained through animal experiments [1]. Considering the high correlation between in vivo and in vitro digestibility [11], the fixed value of in vitro CP digestibility for all AA could serve as an alternative to the SID of AA for calculating DIAAS, as CP digestibility could be highly correlated with AA digestibility. Therefore, the objective of the present work was to compare the DIAAS of various food protein sources calculated using the SID of AA, the IVID_Flask_ of CP, and the IVID_Daisy_ of CP.

The concentrations of CP and AA in white rice, wheat, and SPI used in the present study were within the range of previous studies [6,9,14,22]. The CP concentration in PPC used in the present study was greater than that in previous studies [6,23,24]. The nutrient composition in PPC harvested from different regions may vary due to differences in production environments, genetics, or both, leading to variations in protein concentrations [23,24]. In contrast, the CP concentration in SMP was lower than that in previous studies [6,14]. Although the reason for the relatively low CP concentration in SMP in the present work is unclear, the milking conditions and SMP production processes may have affected the CP concentrations [25,26].

The values for the SID of indispensable AA in white rice, wheat, SPI, and PPC determined in the present work were consistent with those in the previous studies [6,9]. In contrast, the SID of indispensable AA in SMP used in the present work was greater than that in a previous study [6], which may be due to different production technologies, storage conditions, or milking regions for SMP production [6]. Processing can cause denaturation, aggregation, and chemical modifications of AA, potentially altering protein quality [27]. Additionally, prolonged exposure to high ambient temperatures during storage can lead to changes in the milk protein in sterilized or dried dairy products [27].

The negative AID of Pro for white rice has also been observed in other cereal grains with relatively low concentrations of Pro [20,28], which is most likely due to the increased excretion of endogenous losses of Pro when pigs are provided with the feeds containing unbalanced AA [29]. Among the AA, the BEL of Pro was the greatest in the present study, in agreement with previous data [20,28,30]. The large BEL of Pro is mainly attributed to the mucin and enzymes secreted in the ileal digesta. The large BEL of Pro resulted in the SID of Pro being greater than 100% in all ingredients in the present work, which was also observed in the previous studies [20,28,31]. Due to the nature of SID calculation procedures, a large value of BEL results the large SID values [7], particularly for the diets containing a small quantity of AA such as Pro in the present study.

According to the FAO guidelines [1], a food can be considered an excellent- or high-quality protein source when the DIAAS value is greater than 100 and can be considered a good protein source when the DIAAS value is between 75 and 100. In contrast, for food with a DIAAS value below 75, it is not recommended to make any claims regarding protein quality. The DIAAS values of white rice, wheat, PPC, SPI, and SMP were consistent with published DIAAS values for infants, children, and adults [6,9], except for the DIAAS of SMP for infants (82 for Trp in this work vs. 81 for Thr in the literature) [6]. Despite the difference in the DIAAS of SMP between this work and the literature, the SMP used in both studies qualifies as a ‘good’ source of protein for infants from birth to 6 months and an ‘excellent’ source for those older than 6 months. Soy protein isolate is also a ‘good’ source for ages 6 months to older than 3 years, but no claim for infants can be made for SPI. Likewise, no claim for protein quality can be made for white rice, wheat, and PPC because the DIAAS was below 75 for all life stages.

In previous studies, the DIAAS values of grains, including barley, corn, oats, rice, rye, sorghum, and wheat, were reported to be between 29 and 77 [5,6,9], but the DIAAS values of dairy products range from 123 to 142 [6]. The reason for the low DIAAS of grains is that grains have low AA concentrations, particularly Lys concentrations [20,32]. Likewise, the observation that no claim for protein quality can be made for white rice and that the first limiting AA in white rice is Lys is in agreement with previous data [9]. Consuming only such foods with DIAAS values lower than 75 can lead to malnutrition in humans. Therefore, a combination of plant sources such as grains and animal products such as dairy products is needed to provide sufficient AA to fulfill the AA requirements of humans, which can prevent malnutrition [33].

The DIAAS values based on the IVID of CP were similar to those based on the SID of AA for most food protein sources, indicating that both the conventional in vitro procedure and the multi-sample simultaneous in vitro procedure using Daisy^II^ can potentially replace animal experiments for evaluating protein sources for human consumption. Researchers can save time and costs associated with qualifying protein sources by using the quick evaluation system of DIAAS. Although the DIAAS system based on in vitro methods uses CP digestibility instead of AA digestibility, which is conceptually similar to the PDCAAS system, it differs in that in vitro CP digestibility estimates the ileal digestibility of CP, whereas PDCAAS considers the total tract digestibility of CP. The difference may potentially cause confounding effects on nitrogen digestibility due to transamination or deamination resulting from fermentation by microorganisms in the hindgut [5,6]. Additionally, the PDCAAS system is primarily based on data from rats, whereas the in vitro systems were developed to estimate data from pigs [11,16,34]. Therefore, employing in vitro systems is more appropriate for evaluating the protein quality of food protein sources. However, caution is required when evaluating protein quality using in vitro assays, as the classification of protein quality can differ depending on the method used. In addition, the SID values are not the same among the AA of an ingredient. In the present study, the DIAAS of PPC for adults based on the SID of AA was 69, classifying it as ‘no claim’ for protein quality. However, the DIAAS based on the IVID_Daisy_ of CP was 76, classifying it as ‘good’ for protein quality even though the difference in scores was not that much. In contrast, the classifications for white rice, wheat, SPI, and SMP remained consistent across different systems. Further research is warranted to measure the DIAAS of a broader range of food sources for human consumption using both in vivo and in vitro systems.

## 5. Conclusions

In conclusion, white rice, wheat, pea protein concentrate, and soy protein isolate have DIAAS values lower than 100. Therefore, these proteins need to be combined with high-quality protein sources to meet the indispensable AA requirements of humans. In contrast, skim milk powder had high-quality protein, with the DIAAS greater than 100 for children and adults, making it suitable for complementing low-quality protein sources to provide adequate AA in human diets. In addition, in vitro ileal crude protein disappearance can be used to determine the DIAAS of food sources.

## Figures and Tables

**Table 1:** Analyzed nutrient composition of food ingredients (as-fed basis).

Items, %	White Rice	Wheat	PPC	SPI	SMP
Dry matter	87.0	90.5	92.0	95.4	92.0
Gross energy, kcal/kg	3610	3890	4814	5037	3965
Crude protein	7.5	11.6	67.0	84.2	27.6
Ether extract	0.3	3.3	4.2	0.6	ND
Ash	0.8	2.7	6.4	4.4	6.7
Indispensable amino acids					
Arg	0.57	0.62	5.47	6.27	0.98
His	0.17	0.32	1.69	2.21	0.82
Ile	0.25	0.40	3.19	4.02	1.45
Leu	0.53	0.80	5.78	7.07	2.89
Lys	0.25	0.46	4.80	5.16	2.27
Met	0.15	0.19	0.52	0.94	0.61
Phe	0.39	0.56	3.83	4.56	1.39
Thr	0.25	0.41	2.60	3.27	1.29
Trp	0.08	0.13	0.62	1.08	0.40
Val	0.40	0.55	3.43	4.07	1.83
Dispensable amino acids					
Ala	0.37	0.46	3.07	3.76	0.96
Asp	0.61	0.73	7.91	9.59	2.24
Cys	0.18	0.25	0.69	0.86	0.25
Glu	1.21	3.10	11.22	15.96	6.11
Gly	0.30	0.51	2.81	3.54	0.54
Pro	0.25	0.98	3.04	4.50	2.93
Ser	0.37	0.57	3.47	4.33	1.60
Tyr	0.35	0.38	2.37	2.96	1.30

ND = not detected; PPC = pea protein concentrate; SMP = skim milk powder; SPI = soy protein isolate.

**Table 2:** Ingredient composition of experimental diets.

Item, %	White Rice	Wheat	PPC	SPI	SMP	N-Free
White rice	96.8	-	-	-	-	-
Wheat	-	96.9	-	-	-	-
PPC	-	-	25.0	-	-	-
SPI	-	-	-	20.0	-	-
SMP	-	-	-	-	50.0	-
Cornstarch	-	-	50.5	54.0	32.8	67.9
Sucrose	-	-	14.9	15.9	9.7	20.0
Soybean oil	-	-	3.0	3.2	1.9	4.0
Cellulose	-	-	3.0	3.2	1.9	4.0
Ground limestone	0.7	1.3	0.4	0.4	0.4	0.4
Dicalcium phosphate	1.3	0.7	2.1	2.1	2.1	2.1
Chromic oxide	0.5	0.5	0.5	0.5	0.5	0.5
Magnesium oxide	-	-	-	-	-	0.1
Potassium carbonate	-	-	-	-	-	0.4
Sodium chloride	0.4	0.4	0.4	0.4	0.4	0.4
Vitamin and mineral premix ^1^	0.3	0.3	0.3	0.3	0.3	0.3

N = nitrogen; PPC = pea protein concentrate; SMP = skim milk powder; SPI = soy protein isolate. ^1^ Provided the following quantities per kilogram of a complete diet: vitamin A, 18,000 IU; vitamin D_3_, 3600 IU; vitamin E, 60 IU; vitamin K, 4.5 mg; thiamin, 4.5 mg; riboflavin, 7.5 mg; pyridoxine, 4.5 mg; vitamin B_12_, 0.06 mg; pantothenic acid, 30 mg; folic acid, 1.5 mg; niacin, 45 mg; biotin, 0.3 mg; Co, 0.75 mg as cobaltous sulfate; Cu, 60 mg as copper sulfate; Fe, 120 mg as iron sulfate; I, 120 mg as calcium iodate; Mn, 60 mg as manganese sulfate; Se, 0.3 mg as sodium selenite; and Zn, 60 mg as zinc sulfate.

**Table 3:** Analyzed nutrient composition of experimental diets (as-fed basis).

Item, %	White Rice	Wheat	PPC	SPI	SMP	N-Free
Dry matter	87.1	90.7	93.1	94.0	94.2	93.1
Gross energy, kcal/kg	3497	3752	3986	3965	3783	3777
Crude protein	6.9	11.5	16.8	17.2	13.7	ND
Ether extract	0.9	3.7	2.7	1.8	2.4	4.6
Ash	3.3	5.1	4.4	4.2	6.6	3.8
Indispensable amino acids						
Arg	0.58	0.56	1.33	1.28	0.49	ND
His	0.17	0.29	0.43	0.49	0.39	ND
Ile	0.26	0.36	0.77	0.81	0.72	ND
Leu	0.54	0.74	1.38	1.43	1.41	ND
Lys	0.25	0.42	1.20	1.11	1.12	0.01
Met	0.14	0.16	0.15	0.20	0.33	ND
Phe	0.40	0.52	0.95	0.97	0.70	ND
Thr	0.25	0.39	0.65	0.69	0.64	0.01
Trp	0.08	0.12	0.15	0.23	0.20	ND
Val	0.40	0.51	0.88	0.87	0.94	0.01
Dispensable amino acids						
Ala	0.37	0.43	0.73	0.76	0.48	ND
Asp	0.61	0.69	1.96	2.01	1.12	0.02
Cys	0.16	0.24	0.20	0.21	0.15	0.03
Glu	1.24	2.85	2.79	3.37	3.04	0.02
Gly	0.30	0.47	0.69	0.74	0.27	0.01
Pro	0.24	0.95	0.69	0.94	1.40	ND
Ser	0.38	0.53	0.87	0.93	0.80	0.02
Tyr	0.36	0.35	0.58	0.61	0.64	ND

N = nitrogen; ND = not detected; PPC = pea protein concentrate; SMP = skim milk powder; SPI = soy protein isolate.

**Table 4:** Apparent ileal digestibility of crude protein and amino acids in white rice, wheat, pea protein concentrate (PPC), soy protein isolate (SPI), and skim milk powder (SMP).

Item, %	White Rice	Wheat	PPC	SPI	SMP	SEM	*p*-Value
Number of observations	8	9	9	9	9		
Crude protein	71.3 ^b^	73.9 ^b^	83.9 ^a^	87.3 ^a^	84.7 ^a^	1.22	<0.001
Indispensable amino acids							
Arg	86.6 ^c^	82.0 ^d^	92.6 ^a,b^	94.4 ^a^	91.1 ^b^	0.68	<0.001
His	82.0 ^c^	79.6 ^c^	88.3 ^b^	91.1 ^a^	91.7 ^a^	0.78	<0.001
Ile	79.6 ^c^	78.2 ^c^	88.4 ^a,b^	90.1 ^a^	86.8 ^b^	0.76	<0.001
Leu	82.8 ^c^	81.2 ^c^	88.5 ^b^	89.3 ^b^	93.7 ^a^	0.64	<0.001
Lys	72.8 ^c^	72.7 ^c^	89.9 ^b^	91.7 ^a,b^	94.4 ^a^	0.85	<0.001
Met	84.8 ^c^	83.4 ^c^	84.4 ^c^	89.5 ^b^	95.1 ^a^	0.88	<0.001
Phe	85.8 ^c^	82.9 ^d^	89.6 ^b^	91.4 ^a,b^	93.5 ^a^	0.56	<0.001
Thr	66.5 ^b^	67.0 ^b^	80.1 ^a^	81.3 ^a^	82.8 ^a^	1.15	<0.001
Trp	76.2 ^b,c^	71.2 ^c^	78.1 ^b^	86.0 ^a^	89.7 ^a^	1.38	<0.001
Val	76.5 ^c^	71.9 ^d^	82.8 ^b^	84.0 ^ab^	86.5 ^a^	0.93	<0.001
Dispensable amino acids							
Ala	76.3 ^b^	68.2 ^c^	82.1 ^a^	83.2 ^a^	82.8 ^a^	1.33	<0.001
Asp	78.1 ^c^	69.1 ^d^	85.6 ^b^	89.7 ^a^	86.8 ^a,b^	0.95	<0.001
Cys	79.9 ^a^	77.2 ^a,b^	71.6 ^c^	80.7 ^a^	73.1 ^b,c^	1.63	<0.001
Glu	84.2 ^c^	89.6 ^b^	90.9 ^a,b^	92.9 ^a^	88.4 ^b^	0.69	<0.001
Gly	55.2 ^b^	60.9 ^b^	75.3 ^a^	75.7 ^a^	59.9 ^b^	2.92	<0.001
Pro	−39.9 ^b^	67.6 ^a^	63.8 ^a^	67.1 ^a^	93.0 ^a^	11.23	<0.001
Ser	78.0 ^b^	76.6 _b_	85.0 ^a^	87.9 ^a^	74.4 ^b^	1.01	<0.001
Tyr	85.6 ^c^	78.1 ^d^	88.3 ^b^	90.7 ^a,b^	93.3 ^a^	0.68	<0.001

SEM = standard error of the means. ^a–d^ Means within a row without a common superscript letter differ (*p* < 0.05).

**Table 5:** Standardized ileal digestibility of crude protein and amino acids in white rice, wheat, pea protein concentrate (PPC), soy protein isolate (SPI), and skim milk powder (SMP) ^1^.

Item, %	White Rice	Wheat	PPC	SPI	SMP	SEM	*p*-Value
Number of observations	8	9	9	9	9		
Crude protein	96.0 ^a^	89.4 ^b^	94.7 ^a^	96.8 ^a^	98.5 ^a^	1.22	<0.001
Indispensable amino acids							
Arg	96.8 ^b^	93.0 ^c^	97.4 ^b^	99.4 ^b^	104.3 ^a^	0.68	<0.001
His	94.0 ^b^	87.0 ^c^	93.3 ^b^	95.6 ^a,b^	97.3 ^a^	0.78	<0.001
Ile	90.0 ^b^	85.8 ^c^	92.1 ^a,b^	93.7 ^a^	90.8 ^a,b^	0.76	<0.001
Leu	91.8 ^b^	88.0 ^c^	92.2 ^b^	92.9 ^b^	97.4 ^a^	0.64	<0.001
Lys	86.7 ^c^	81.2 ^d^	93.0 ^b^	95.0 ^a,b^	97.7 ^a^	0.85	<0.001
Met	90.4 ^c^	88.7 ^c^	90.0 ^c^	93.7 ^b^	97.7 ^a^	0.88	<0.001
Phe	93.7 ^b^	89.2 ^c^	93.2 ^b^	95.0 ^b^	98.5 ^a^	0.56	<0.001
Thr	90.9 ^a^	83.5 ^b^	90.1 ^a^	90.8 ^a^	93.1 ^a^	1.15	<0.001
Trp	91.8 ^ab^	82.2 ^c^	87.1 ^b,c^	92.1 ^a^	96.6 ^a^	1.38	<0.001
Val	92.6 ^a^	85.0 ^b^	90.6 ^a^	92.0 ^a^	93.9 ^a^	0.93	<0.001
Dispensable amino acids							
Ala	91.1 ^a,b^	81.6 ^c^	90.1 ^b^	90.9 ^a,b^	95.2 ^a^	1.33	<0.001
Asp	90.9 ^b,c^	81.1 ^d^	89.9 ^c^	94.0 ^a,b^	94.5 ^a^	0.95	<0.001
Cys	96.4 ^a^	88.5 ^b,c^	86.1 ^c^	94.1 ^a^	92.6 ^a,b^	1.63	<0.001
Glu	92.3 ^b^	93.3 ^b^	94.7 ^a,b^	96.1 ^a^	91.9 ^b^	0.69	<0.001
Gly	111.3 ^b^	98.3 ^c^	101.4 ^b,c^	100.4 ^b,c^	126.9 ^a^	2.92	<0.001
Pro	191.9 ^a^	129.5 ^b^	151.3 ^a,b^	132.2 ^b^	136.6 ^b^	11.23	0.001
Ser	94.7 ^a^	89.1 ^b^	92.8 ^a,b^	95.3 ^a^	83.0 ^c^	1.01	<0.001
Tyr	93.9 ^b^	87.1 ^c^	93.9 ^b^	96.0 ^a,b^	98.3 ^a^	0.68	<0.001

SEM = standard error of the means. ^1^ Standardized ileal digestibility of amino acid was calculated by correcting the apparent ileal digestibility of amino acid for the basal endogenous losses of amino acid. Values used for the basal endogenous losses were as follows (g/kg of dry matter intake): crude protein, 19.63; Arg, 0.68; His, 0.23; Ile, 0.30; Leu, 0.55; Lys, 0.40; Met, 0.09; Phe, 0.37; Thr, 0.70; Trp, 0.15; Val, 0.74; Ala, 0.63; Asp, 0.91; Cys, 0.30; Glu, 1.15; Gly, 1.94; Pro, 6.49; Ser, 0.73; Tyr, 0.35. ^a–d^ Means within a row without a common superscript letter differ (*p* < 0.05).

**Table 6:** In vitro ileal disappearance of crude protein (CP) in white rice, wheat, pea protein concentrate (PPC), soy protein isolate (SPI), and skim milk powder (SMP) ^1^.

Item, %	White Rice	Wheat	PPC	SPI	SMP	SEM	*p*-Value
IVID_Flask_ of CP	95.8 ^a^	88.5 ^d^	91.0 ^c^	93.8 ^b^	97.0 ^a^	0.42	<0.001
IVID_Daisy_ of CP	88.5 ^c^	90.3 ^c^	97.2 ^b^	99.9 ^a^	100.0 ^a^	0.52	<0.001

IVID_Flask_ = in vitro ileal disappearance based on the conventional in vitro assay using flasks; IVID_Daisy_ = in vitro ileal disappearance based on the multi-sample simultaneous in vitro procedure using the Daisy^II^ incubator (ANKOM Technology, Macedon, NY, USA); SEM = standard error of the means. ^1^ Each mean represents three observations. ^a–d^ Least squares means with a row without a common superscript letter differ (*p* < 0.05).

**Table 7:** Digestible indispensable amino acid scores (DIAAS, %) for white rice, wheat, pea protein concentrate (PPC), soy protein isolate (SPI), and skim milk powder (SMP) ^1^.

Item	White Rice	Wheat	PPC	SPI	SMP
DIAAS in infants ^2^					
SID of AA	42 (Lys)	46 (Lys)	47 (Trp)	61 (SAA)	82 (Trp)
IVID_Flask_ of CP	46 (Lys)	50 (Lys)	49 (Trp)	61 (SAA)	82 (Trp)
IVID_Daisy_ of CP	42 (Lys)	51 (Lys)	53 (Trp)	65 (SAA)	85 (Trp)
DIAAS in children ^3^					
SID of AA	50 (Lys)	56 (Lys)	58 (SAA)	74 (SAA)	112 (SAA)
IVID_Flask_ of CP	56 (Lys)	61 (Lys)	60 (SAA)	74 (SAA)	112 (SAA)
IVID_Daisy_ of CP	51 (Lys)	62 (Lys)	65 (SAA)	79 (SAA)	116 (SAA)
DIAAS in adults ^4^					
SID of AA	60 (Lys)	66 (Lys)	69 (SAA)	87 (SAA)	131 (SAA)
IVID_Flask_ of CP	66 (Lys)	72 (Lys)	71 (SAA)	87 (SAA)	132 (SAA)
IVID_Daisy_ of CP	61 (Lys)	74 (Lys)	76 (SAA)	93 (SAA)	136 (SAA)

AA = amino acids; AAA = aromatic amino acids (Phe + Tyr); CP = crude protein; IVID_Flask_ = in vitro ileal disappearance based on the conventional in vitro assay using flasks; IVID_Daisy_ = in vitro ileal disappearance based on the multi-sample simultaneous in vitro procedure using the Daisy^II^ incubator (ANKOM Technology, Macedon, NY, USA); SAA = sulfur amino acids (Met + Cys); SID = standardized ileal digestibility. ^1^ Digestible indispensable AA-to-AA in the reference protein ratio (%) was calculated by dividing the standardized ileal digestible AA concentration (mg/g CP) by the AA concentration (mg/g CP) in the reference protein for each age range. The DIAAS (%) was the lowest value of digestible AA-to-AA in the reference protein ratio. ^2^ The AA concentrations (mg/g CP) in the reference protein for infants (birth to 6 months) are as follows: His, 21; Ile, 5; Leu, 96; Lys, 69; SAA, 33; AAA, 94; Thr, 44; Trp, 17; Val, 55. ^3^ The AA concentrations (mg/g CP) in the reference protein for children (6 months to 3 years) are as follows: His, 20; Ile, 32; Leu, 66; Lys, 57; SAA, 27; AAA, 52; Thr, 31; Trp, 8.5; Val, 43. ^4^ The AA concentrations (mg/g CP) in the reference protein for adults (older than 3 years) are as follows: His, 16; Ile, 30; Leu, 61; Lys, 48; SAA, 23; AAA, 41; Thr, 25; Trp, 6.6; Val, 40.

## Data Availability

The data presented in the current work are available.
